# Insights into radiomics: a comprehensive review for beginners

**DOI:** 10.1007/s12094-025-03939-5

**Published:** 2025-05-12

**Authors:** Francesco Mariotti, Andrea Agostini, Alessandra Borgheresi, Marzia Marchegiani, Alice Zannotti, Gloria Giacomelli, Luca Pierpaoli, Elisabetta Tola, Elena Galiffa, Andrea Giovagnoni

**Affiliations:** 1 Department of Clinical, Special and Dental Sciences, University Politecnica delle Marche, Via Tronto, 10/A, 60126 Ancona, Italy; 2 Department of Radiological Sciences - Division of Clinical Radiology, University Hospital “Azienda Ospedaliero Universitaria delle Marche”, Via Conca, 71, 60126 Ancona, Italy; 3 School of Radiology, University Politecnica delle Marche, Via Tronto, 10/A, 60126 Ancona, Italy

**Keywords:** Radiology, Oncology, Radiomics, Artificial intelligence, Digital health

## Abstract

Radiomics and artificial intelligence (AI) are rapidly evolving, significantly transforming the field of medical imaging. Despite their growing adoption, these technologies remain challenging to approach due to their technical complexity. This review serves as a practical guide for early-career radiologists and researchers seeking to integrate radiomics into their studies. It provides practical insights for clinical and research applications, addressing common challenges, limitations, and future directions in the field. This work offers a structured overview of the essential steps in the radiomics workflow, focusing on concrete aspects of each step, including indicative and practical examples. It covers the main steps such as dataset definition, image acquisition and preprocessing, segmentation, feature extraction and selection, and AI model training and validation. Different methods to be considered are discussed, accompanied by summary diagrams. This review equips readers with the knowledge necessary to approach radiomics and AI in medical imaging from a hands-on research perspective.

## Introduction

In recent years, radiomics has experienced a significant increase in scholarly interest, evidenced by a notable annual growth rate of 200% of scientific production from 2017 to 2023 [1]. This growth is particularly remarkable, rising sharply from 120 publications in 2017 to 1500 in 2023. About 45.8% of these publications belong to the medical domain, with a main focus on radiological studies such as radiomics in radiology, nuclear medicine, and medical imaging (RNMMI) [2]. These studies often focus on oncology, positioning radiomics as an additional valuable tool for supporting diagnosis.

**Fig. 1:**
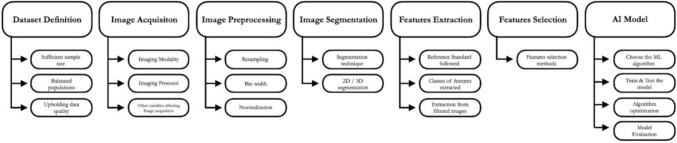
Diagram of the main steps in a radiomics study

Radiomics, exploiting quantitative analysis on medical images, extracts a set of high-dimensional data driving a significant change in diagnostic and prognostic methods. The idea behind radiomics is that biomedical images encapsulate intricate details of disease-related phenomena [3]. These details often escape human perception and are not accessible through conventional visual inspection of the images. Using mathematical algorithms to analyze patterns in signal intensities and pixel relationships, radiomics aims to measure the textural features [4]. While traditional biomarker development typically begins with biology-based hypotheses, the development of radiomic biomarkers (textural features) is data driven. Methods such as genomics, transcriptomics, proteomics, and radiomics are employed to explore huge datasets in search of sensitive markers for predicting outcomes, often leading to the generation of post hoc hypotheses [5]. Nowadays, the merging of radiomics and artificial intelligence has generated significant interest among physicians, marking a new way to approach diagnostics and research methods. A narrative review is chosen as it provides context and background information on radiomics in medical radiology, addressing broader questions related to key developments, methodologies, and challenges in this field. This narrative review aims to support physicians delving into radiomic research (specifically tailored to medical radiology) and defines the essential steps to conduct a reliable radiomics study. Figure 1 schematically represents each step to be accounted, while Table 1 provides a practical example.

**Table 1:** Example study: magnetic resonance (MR) radiomics-based diagnosis of hepatocellular carcinoma (HCC) in chronic liver disease (CLD) patients

Step	Key question	Management	
Dataset definition	Is there a sufficient sample size?	Identified 104 patients with CLD under screening for the risk of HCC development	
	Is the population balanced?	54 patients affected by HCC (Group 1—histologically confirmed) and 50 patients with no lesions HCC (Group 0)	
Image acquisition	What is the imaging modality used?	All patients undergo a 1.5 T liver MR scan	
	What is the scanner used?	Two scanners from different vendors are used	
	What is the protocol used?	All patients are examined with the same standardized MR liver protocol	
Image preprocessing	Is normalization performed?	Normalization is applied to ensure consistency in intensity values	
	Is resampling applied?	Resampling is performed to achieve uniform voxel spacing across all images (1 × 1 × 1 mm^3^)	
	Is fixed bin width used?	Fixed bin width is used for intensity discretization, ensuring consistency in following texture feature extraction (25)	
Image segmentation	How is segmentation performed?	Semi-automatic segmentation performed by two radiologists in consensus	
	Is it 2D or 3D segmentation?	3D full liver segmentation	
Feature extraction	Is feature extraction based on a reference standard?	Features are extracted according to PyRadiomics standards	
	Which classes of features are extracted?	All feature classes defined by PyRadiomics are included	
	Are features extracted from filtered images?	Features are extracted from original, wavelet-filtered, and logarithmic-filtered images	
Features selection	How is feature selection performed?	From 1070 extracted features, the 10 most relevant ones are selected using the minimum redundancy maximum relevance (mRMR) algorithm	
AI model	Which AI model is chosen?	A machine learning random forest model with 100 trees, maximum depth 10, minimum samples per split 5, minimum samples per leaf 3	
	How is the dataset split?	Stratified train test validation split 70–20-10%	
	What metrics are used to evaluate the model?	Accuracy, F1-score, and AUC are used as model evaluation metrics	

### Dataset definition

A solid radiomics study begins with a clear goal and a well-defined patient population [6]. There are three cardinal rules in defining a dataset:

- Ensuring sufficient sample size.
- Achieving balanced representation across patient populations.
- Upholding data quality.

An adequate sample size is crucial to provide useful information to the model (enabling it to capture complex patterns and relationships within the data) and to avoid overfitting [7]. The right sample size could be evaluated based on the rule of thumb, such as the sample size needs to be at least 50 times the number of prediction classes and/or the sample size needs to be at least 10 times the number of the selected features [8].

A dataset composed of two or more patient populations, depending on the number of targets considered in the model, is balanced when these populations have comparable sample sizes [9]. In clinical studies, the most significant imbalances between populations practically occur when analyzing rare but crucial occurrences. In these cases, efforts should be addressed to maximize the balance between the populations under analysis. Usually, two different approaches are employed: undersampling the greater population, for data reduction, or oversampling the smaller population, for data augmentation [10]. Generally, the major drawback of undersampling techniques is the discarding of potentially useful data [11]. Despite this limitation, undersampling seems to be the most correct approach in managing significantly imbalanced datasets [12]. However, considering the data scarcity issue in the medical scenario, there is a prevalent inclination to employ oversampling techniques when dealing with imbalanced datasets [13]. The limitation of oversampling relies on its tendency to create subjects that are not significantly divergent from the original ones, thereby amplifying any biases already present in the initial population [14]. Moreover, oversampling techniques tend to cause overperformance in the model since the hybrid subjects are not excessively dissimilar from the originals, and the model deviates from the observed reality [15].

Lastly, it is essential to ensure data quality, using consistent protocols on the same equipment under standardized conditions [16]. More details are provided in the following paragraphs.

### Image acquisition

The radiomic information can be derived from bioimages acquired through two-dimensional or volumetric acquisitions using various techniques, such as X-rays, MRI, nuclear medicine, ultrasounds, or dose maps obtained in radiotherapeutic plans [17]. Radiomic data depend on the imaging technique and acquisition settings, which can be both a strength and a limitation [18]. To conduct a radiomics study effectively, it is preferable for the images used to meet three fundamental conditions:

- They should all originate from the same modality and preferably form the same scanner.
- The acquisition protocol should be consistent and standardized across all the images [19].
- The impact of all other variables and external conditions that may influence acquisition should be minimized or at least kept consistent across all images [20].

Radiomic features are highly sensitive to variations in imaging modality, protocol, and reconstruction parameters, which can obscure the biological aspects [21]. To address this challenge the Radiological Society of North America and the National Institute for Biomedical Imaging and Bioengineering have introduced initiatives like the Quantitative Imaging Biomarkers Alliance (QIBA) and the European Imaging Biomarkers Alliance subcommittee (EIBALL) [22]. These groups reached a consensus on the measurement accuracy of quantitative imaging biomarkers and outline requisite procedures for achieving optimal accuracy levels.

It is strongly recommended to define a preprocessing step in the pipeline of a radiomic project to analyze medical images. This preprocessing phase is functional in ensuring dataset uniformity and consistency, thereby fortifying the robustness and reliability of subsequent analyses [23].

### Image preprocessing

Data quality is closely linked to radiomic features repeatability and reproducibility ("Garbage In, Garbage Out") [24]. These features may be influenced, for example, by the quality of the input images determined by multiple factors related to image acquisition. These factors include scanner equipment, acquisition techniques, reconstruction parameters, and contrast administration, among others [20]. Detailed and complete discussion of image preprocessing is intricate and extensive and is beyond the scope of this review and is extensively discussed elsewhere [25]. Here, the main preprocessing steps, for accurately handling radiological images before extracting radiomic features, are discussed [26]. Within the preprocessing pipeline for radiological image analysis, there are three main steps to be considered:

- Resampling.
- Bin width setting.
- Normalization.

These steps play an important role in ensuring the comparability and interpretability of the feature extraction process.

**Fig. 2:**
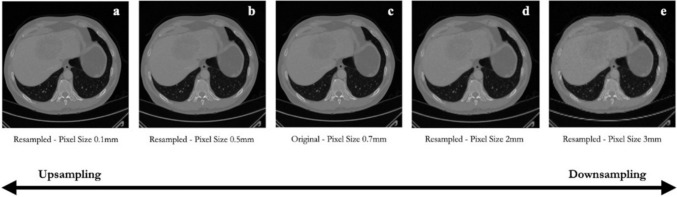
Effect of resampling: upsampling and downsampling on the same test image. Images **a** and **b**, obtained through upsampling. Images **d** and **e**, obtained through downsampling. **c** is the original image

Resampling involves the alteration of the spatial resolution of an image, thereby mitigating the potential differences resulting from the variations in acquisition devices or protocols [27]. Through resampling, a uniform grid is established, enhancing the standardization of radiological images and facilitating subsequent analyses [22]. In the study conducted by Yao F et al. [28], a uniform grid of 2 × 2 × 2 mm^3^ is proposed. The focus of this study is on PET images, and a larger voxel size (compared to CT) is preferable for statistical reasons. The utilization of this resampling strategy contributes to achieving a more reliable statistical representation of the radiotracer uptake and distribution in the prostate, facilitating the subsequent machine learning-based prediction of diverse biological characteristics associated with multiple primary prostate cancers. On the contrary, Levi R et al. [29] adopted a different resampling approach, specifically a uniform grid of 0.3 × 0.3 × 0.3 mm^3^. Their study focuses on the physiological modifications of the bone structure, and smaller voxel dimensions are necessary to find subtle variations related to age and sex. The different resampling strategies proposed in these studies highlight the absence of a predefined optimal configuration, which instead depends on factors such as the study objectives, anatomical structures under analysis, and imaging techniques employed [30]. Figure 2 shows the effect of upsampling and downsampling on a liver image.

**Fig. 3:**
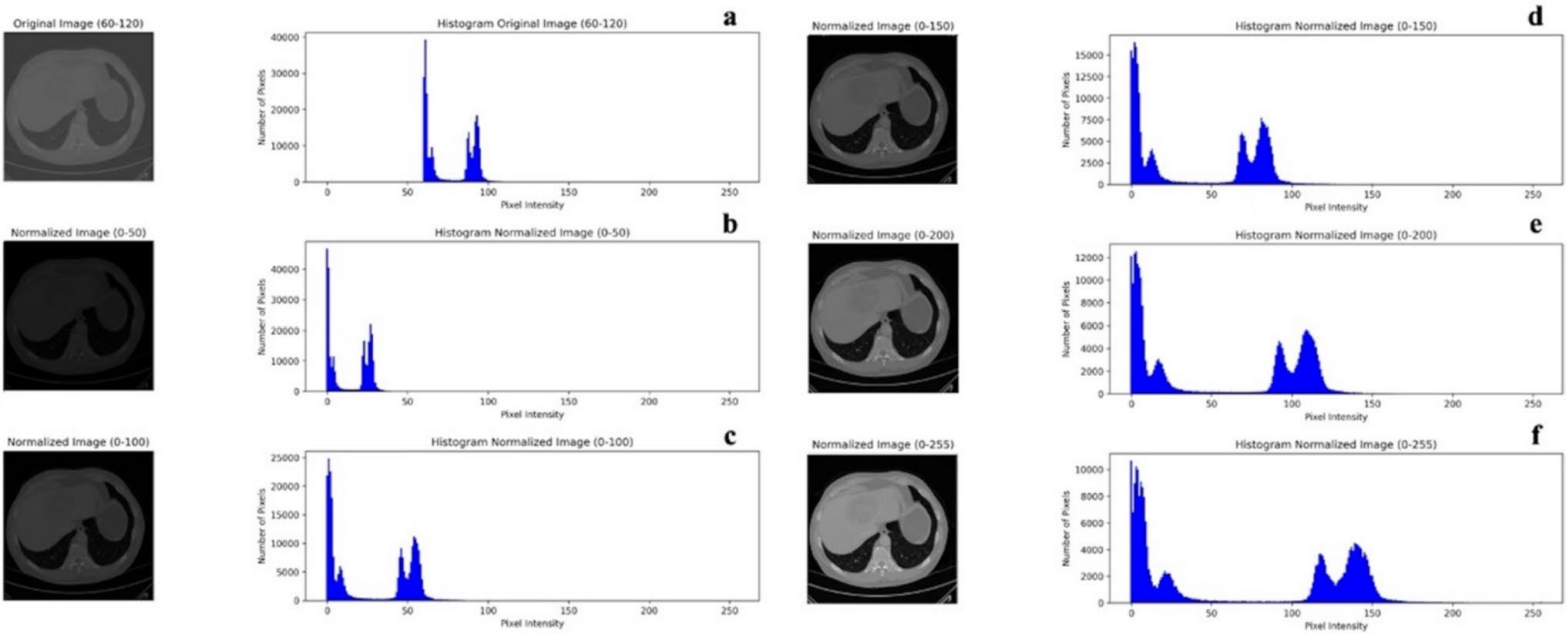
Demonstration of image normalization applied to an original CT scan image with pixel intensities ranging from 60 to 120. The histograms and images show the effects of normalization across different pixel intensity ranges. **a** Original image (60–120), **b** normalized image (0–50), **c** normalized image (0–100), **d** normalized image (0–150), **e** normalized image (0–200), and **f:** normalized image (0–255). The process illustrates how normalization alters the distribution of pixel intensities, enhancing image contrast and visibility

Bin width setting represents the definition of intervals (bins) into which pixel intensity values are grouped [31]. This process influences how well small intensity variations are captured in radiological data. Selecting an appropriate bin width, for pixel intensity discretization, is a critical decision that depends on several factors, including data characteristics, analysis objectives, and sensitivity to intensity variations [32]. The choice of bin width must ensure that the main peculiarities of the distribution are preserved without introducing significant bias. As suggested by Van Griethuysen J. et al. [33], a number of bins ranging between 30 and 130 can be considered adequate in most cases. Defining the optimal bin width a priori presents a challenge, for instance, Van de Berg R. et al. [34] employ a fixed bin width of 0.5 in their study on automated diagnosis of Menière’s disease, while Zhang J. et al. set different bin widths according to the imaging technique employed [31]. Alternatively, an intriguing approach proposed by Scarsbrook A. et al. [35] involves adopting a fixed bin count strategy [36]. Normalization concerns the standardization of pixel intensity values across radiological images [37]. It aims to establish a standardized scale, which improves the comparability and interpretability of subsequent quantitative analyses. In cases involving heterogeneous data from different machines and different acquisition protocols, normalization is necessary. Addressing this limitation is essential for ensuring the validity and generalizability of the radiomic features across different cohorts and imaging conditions. For instance, Gao W. et al. [38] strategically employed linear normalization to reconcile images obtained from distinct scanners operating at 1.5 Tesla (1.5 T) and 3.0 Tesla (3.0 T) [38]. Figure 3 shows the same images with different normalization scales.

### Image segmentation

Segmentation is a crucial step in radiomic studies, though often underestimated and prone to errors [39]. While developing a radiomics study, it is fundamental to decide how to perform segmentation by defining two main aspects:

- Whether to perform segmentation automatically, semi-automatically, or manually,
- Whether to segment regions of interest (ROIs) or volumes of interest (VOIs).

The process of image segmentation may be conducted manually, semi-automatically (region growing or thresholding), or automatically [40].

Manual segmentation has several drawbacks, notably being time-consuming and strongly observer biased; therefore, to mitigate these issues, at least two experienced operators perform the segmentation reaching a consensus. The strength of a radiomic study lies in its reproducibility and its independence from minor differences in segmentation introduced by different operators [41]. Therefore, it is advisable to establish a standardized and accurate process for manual segmentation that could mitigate intra-operator and inter-operator variability [42].

Semi-automatic methods such as region growing or thresholding are a valuable alternative. They often require physician intervention in the initial phase to guide the segmentation process, followed by automatic pixel classification based on predefined criteria [43]. While these methods speed up the segmentation process, they also require physician final refinement to check for coarse segmentation and insufficient precision in the results.

Automatic segmentation handles the issue of reproducibility; for example, Zhen et al. propose an automatic deep learning-based segmentation to overcome oncologist variability in clinical target volume (CTV) segmentation [44]. However, automatic segmentation also hides some pitfalls, since the generalizability of automatic segmentation algorithms is not straightforward. These algorithms perform well on the dataset they were developed with, but they can lead to complete failure when applied to other datasets [45]. Therefore, additional research efforts must be directed toward developing robust and generalizable algorithms for automated image segmentation.

Defining ROIs or VOIs is a crucial step in image segmentation, as they set the boundaries where radiomic features are calculated [31, 46]. Choosing between them depends on several factors, such as:

- the type and quality of available images,
- the research objective,
- the segmentation technique used.

There are no a priori general conditions that can determine which of the two approaches is superior to the other and depends on the study aim and design. In general, VOI segmentation is more time consuming, while ROI segmentation provides more limited information [18]. In oncological applications, for example, one might choose to segment the lesion on a single slice (ROI) or in its entirety (VOI), depending on the clinical and research objectives.

### Features extraction

Following segmentation, radiomic feature extraction quantifies grayscale characteristics within ROIs or VOIs [47]. In this phase, it is fundamental to ensure the following:

- The establishment of a reference standard for radiomic feature extraction [48].
- Definition of the classes of radiomic features to be extracted [48].
- Evaluating whether to extract radiomic features from the original image and/or from post-processed images [48].

Since there are various methods for the calculation of radiomic features, it is advisable to refer to recognized and authoritative guidelines or standards such as IBSI and PyRadiomics [33].

Furthermore, radiomic features can be categorized into different classes such as:

- First order: statistics calculated from the intensity histogram of the image.
- Shape: features describing the shape and size of the ROI.
- Gray level co-occurrence matrix (GLCM): texture features derived from the spatial arrangement of pixel intensities.
- Gray level run length matrix (GLRLM): texture features based on the number of consecutive pixels with the same intensity (run).
- Gray level size zone matrix (GLSZM): texture features characterizing the size zones of homogeneous intensity regions.
- Gray level dependence matrix (GLDM): texture features based on the dependence between pairs of pixels.
- Neighboring gray tone difference matrix (NGTDM): texture features capturing the difference between the intensity of a pixel and its neighbors.

Features belonging to more complex classes are often more informative but also less reproducible, and this could indicate their lower robustness [18]. For example, Thomas et al. [49], in their study on reproducibility in radiomic features on CT acquisitions, found that first-order features are more reproducible than shape metrics and texture features. Gitto et al. [50] adopted an intriguing approach by conducting a stability analysis as the first step in the feature selection process excluding non-stable ones. Secondly, they selected a subset of the remaining features which maximizes AI model performances. Therefore, it is important in radiomics studies to carefully assess the trade-off between feature complexity (information content) and reproducibility (robustness) based on specific research objectives and clinical applications [51].

In a radiomics study, it is also important to choose whether to extract all the possible features or to select those belonging to specific classes [52]. In the first case, the maximum amount of information is obtained however a larger volume of data implies a higher computational cost. On the other hand, optimization of the feature extraction is performed collecting radiomic features only from certain classes [53]. This will reduce the computational cost and possibly will not affect the information obtained [49].

The radiomic features can be extracted not only from the original preprocessed images [54], but also after applying filters such as:

- Wavelet: produces eight decompositions per level.
- LoG (Laplacian of Gaussian): an edge enhancement filter that highlights areas of gray level change.
- Square: computes the square of the image intensities and then scales them linearly back to the original range.
- SquareRoot: computes the square root of the absolute image intensities and scales them back to the original range.
- Logarithm: applies the logarithm to the absolute intensity.
- Exponential: applies the exponential function to the absolute intensity.

**Fig. 4:**
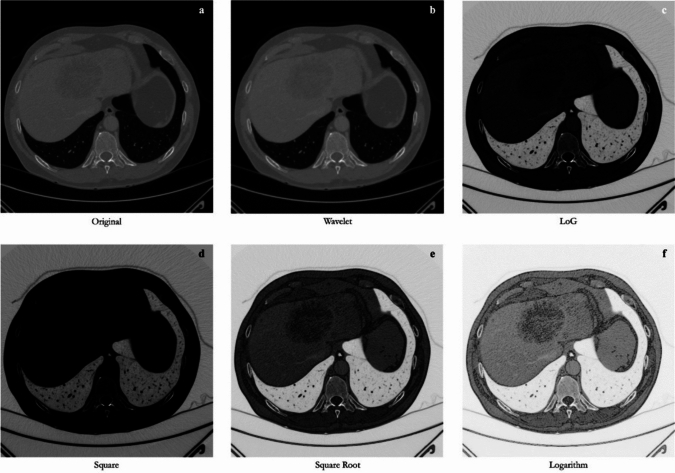
Application of different filters. **a** Original image. **b** Wavelet filtered. **c** Laplacian of Gaussian filtered. **d** Square filtered. **e** Square root filtered. **f** Logarithm filtered

These filers may highlight some aspects of the radiological image, thereby changing their textural values. Potentially filtered images can provide more informative radiomic features than unfiltered images [55]. The extraction of features both from the original and filtered images creates a larger set of features that includes redundant or irrelevant information [56]. An example of filter application in a CT image of focal liver lesion is shown in Fig. 4.

### Features selection

Radiomic features are used as predictors in machine learning (ML) models. A very high number of features can be extracted from radiological images. If all features were used to train an ML model, it would lead to overfitting. Overfitting happens when the model is too complicated for the amount and types of data it is trained on, causing it to closely match the training data [57]. As a result, an overfitted model may perform well on training data, but poorly on new, unseen data. For this reason, it is necessary to select only certain features to create the ML model. There is no unique rule for choosing the appropriate number of radiomic features to use; literature review shows that most follow rules of thumb such as having the number of selected features to be less than 1/10 of the data in the dataset [58].

Features selection methods can be categorized into three groups:

- Filter methods: these methods assess features based on statistical properties; they filter out irrelevant or redundant features before model training [59].
- Wrapper methods: typically involve iterative features selection processes that test different combinations of features to identify the subset that optimizes model performance [60].
- Embedded methods: in these methods, features selection is performed as part of the model optimization, with the objective of selecting the most relevant features while training the model [61].

For a more detailed explanation of these methods, please refer to the material published by Stańczyk U [62].

Features selection is an indispensable step in conducting a correct radiomic study that reduces overfitting and data complexity, improves interpretability and model performance and, last but not least, optimizes computational resources [63].

### AI model

The final step of a radiomics study is the development of a predictive ML model based on the selected radiomic features [64]. The following points have to be taken into account:

- What is the most suitable ML algorithm [65]?
- How to train and test the model.
- How to optimize the algorithm's parameters [66].
- How to evaluate the model [67].

It is not possible to define a priori which is the most suitable algorithm for the dataset considered.

Firstly, it is necessary to decide whether to proceed with supervised or unsupervised learning [68]. In unsupervised training, data is provided to the model without labels, meaning the model does not know which classes the data belong to. The goal of these models is to discover patterns among the dataset that maximize the separation of data into one or more clusters (groups of hypothetical similar or related objects) [69]. Unsupervised learning is better suited to exploring and understanding data structure, rather than predicting specific outputs. Most of the unsupervised algorithms are employed for clustering because of the absence of predefined outcomes and the diversity of the data [70]. Despite their utility and efficiency, these unsupervised methods are unpopular in healthcare studies [71].

On the contrary, in supervised training, the focus is on developing a model capable of interpreting data to predict the targets defined by physicians [72]. For this reason, supervised learning is typically preferred in radiomic medical studies.

Examples of supervised learning models include:

- Logistic regression: linear model used for binary classification tasks [73].
- Support vector machines: identify the optimal hyperplanes to separate data into different classes.
- Random forest: ML models that build multiple decision trees and aggregate their predictions, offering high accuracy and resilience to overfitting.
- Deep neural networks: composed of multiple layers of interconnected neurons, capable of learning intricate patterns and representations from data for classification and regression.

Model choice depends on dataset size and problem complexity [74]. Deep neural networks require large datasets to avoid overfitting, while simpler models suit smaller datasets [75].

**Fig. 5:**
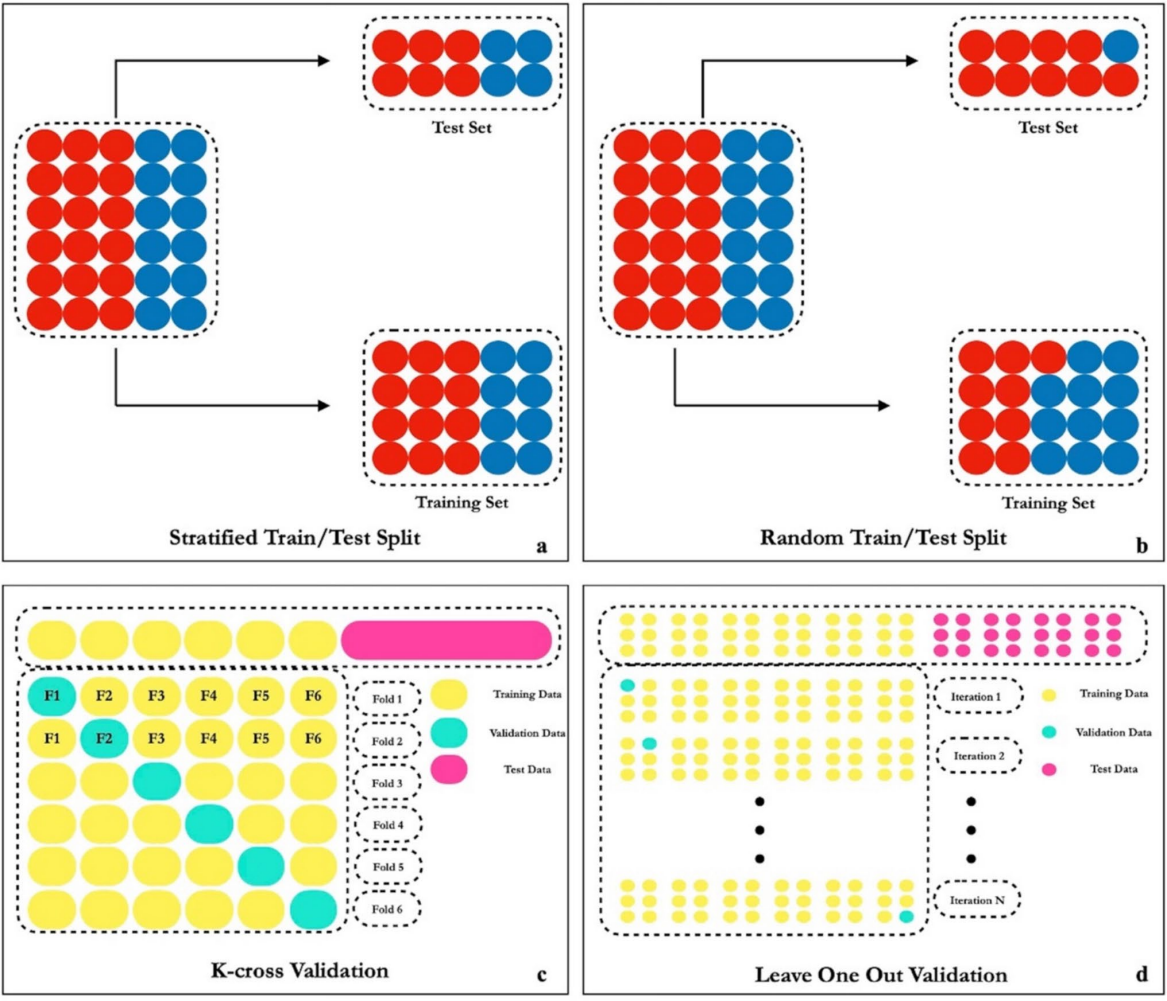
Examples of possible train/test split technique. **a** Stratified train/test split, the dataset is divided into the training and test sets while preserving the proportion of different classes. **b** Random train/test split, the dataset is divided randomly into the training and test sets without ensuring class proportion preservation. **c** K-cross validation, the dataset is divided into K subsets (folds). Each fold is used once as a validation set while the remaining K-1 folds form the training set. This process is repeated K times. **d** Leave one out validation, each data point is used once as a validation set, while the remaining data points form the training set. This process is repeated for each data point in the dataset

To proceed with the training and testing of the model, it is necessary to first divide the dataset into a training subset and a testing subset [76]. This step is mandatory because testing cannot be conducted on data already seen by the model during training [77]. Various approaches exist for the train–test split [78], such as (Fig. 5):

- Stratified train/test split: dataset splitting is performed without changing the proportion of classes under analysis respecting also the desired splitting percentage (usually 80% training, 20% testing) [79].
- Random train/test split: only the percentage of splitting between training and testing is defined [80].
- K-cross validation: splitting the dataset into multiple subsets, training the model on several combinations of these subsets and testing it, for each subset combination on the one left out during training [81].
- Leave one out: special case of K-fold cross-validation where the number of folds equals the number of data points in our dataset [82].

The chosen algorithm needs to be fine-tuned with the optimization of its specific parameters [66]. This is a complex and time-consuming phase that often requires multiple iterations. There are several techniques, including:

- Adjust hyperparameters: manually tuning the parameters of the model, such as learning rate, batch size, or number of layers, to optimize performance [83–85].
- Grid search or random search: methods used to systematically explore different combinations of hyperparameters [86].
- Regularization techniques: prevent overfitting by adding a penalty term (to the loss function); this penalty discourages the model from fitting the training data too closely and helps improve generalization performance [87].

After completing the training and testing phases, there are several metrics that can indicate the quality of the developed model [88]. Among the most commonly used ones, there are:

- Accuracy: The percentage of correct predictions out of the total predictions made by the model. This metric is common for classification problems [89].
- Recall (sensitivity or true positive rate): The percentage of actual positive instances correctly identified by the model out of all actual positive instances [90]. It is useful when capturing the maximum number of positives is important, even at the cost of some false positives.
- Precision: The percentage of actual positive instances correctly identified by the model out of all instances identified as positive by the model [91]. This metric is relevant when it is important to minimize false positives.
- F1-score: A harmonic mean of precision and recall. It is useful when you want to balance precision and recall [92].
- Confusion matrix: A table that shows the number of correct and incorrect predictions made by the model in a classification problem. It is useful for gaining insights into the model's performance [93].
- Receiver operating characteristic (ROC) curve and area under the Curve (AUC): Used primarily for binary classification problems, these metrics evaluate the model's performance by considering the trade-off between true positive rate and false positive rate [94].
- Mean absolute error (MAE): The average of the absolute differences between the model's predictions and the observed values in the test data. It is a common metric for regression problems [95].
- Mean squared error (MSE): The average of the squared differences between the model's predictions and the observed values [95].

Having good results in these metrics does not guarantee the model's generalizability, but simply indicates its performance relative to the dataset used [96]. The ability of a model to generalize indeed depends on many factors that metrics cannot evaluate:

- The model's generalization capability is related to the diversity and representativeness of the dataset used for training and testing.
- Overly good values in the metrics may indicate that the model has adapted too closely to the specific data (overfitting) or is not complex enough to capture the relationships between the data (underfitting) [97].
- In terms of equally obtained metrics, models generated from larger initial datasets tend to generalize better because they learn from a larger statistical sample.

## Conclusions

Radiomics is a valuable quantitative tool for analyzing medical images, revealing features that are often imperceptible through traditional visual inspection. This comprehensive review outlines the radiomics pipeline, emphasizing crucial steps such as dataset definition, image acquisition, preprocessing, segmentation, feature extraction and selection, and AI model development. By addressing key practices and potential pitfalls, this review aims to guide beginners through each step of a radiomic study, answering essential questions that arise throughout the process. The analysis of the literature indicates a gradual convergence toward a common understanding of the correct methodological approach in radiomic research. Although no single, universally correct approach exists, as some methodological choices remain context dependent, efforts to develop standardized frameworks for radiomic studies are already underway. Continued refinement and widespread adoption of these frameworks are expected to further minimize potential inconsistencies. The observed trend suggests that future generations of radiologists and researchers will need to develop a solid understanding of radiomics fundamentals, as the integration of artificial intelligence and radiomics into clinical practice is expected to become increasingly prevalent.

## Data Availability

Not applicable.
